# Patient satisfaction following gender affirming facial surgery: a GENDER-Q and gender preoccupation and stability questionnaire study (GPSQ)

**DOI:** 10.1186/s41687-026-01016-1

**Published:** 2026-05-04

**Authors:** Helia C. Hosseini, Thayer J. Mukherjee, Paula Flores-Perez, Melanie Vassallo, Jacqueline Ihnat, Sasha Wood, Kevin Hu, Neil Parikh, Omar Allam, Michael Alperovich

**Affiliations:** Division of Plastic and Reconstructive Surgery, Department of Surgery, Yale School of Medicine, 330 Cedar Street, Boardman Building, New Haven, Connecticut USA

**Keywords:** Gender-affirming facial surgery, Transgender health, Patient-reported outcomes, Gender dysphoria, GENDER-Q

## Abstract

**Background:**

Gender-affirming facial surgery (GAFS) addresses gender dysphoria by aligning facial features with gender identity, with historical emphasis on refining the upper facial third to render patients more attractive and feminine. The GENDER-Q, a novel patient-reported outcome measure (PROM), evaluates aesthetic, functional, and psychosocial outcomes, complementing the Gender Preoccupation and Stability Questionnaire (GPSQ) to assess effects of GAFS in this study.

**Methodology:**

Survey data from 86 patients was analyzed using GENDER-Q and GPSQ scores. Higher GENDER-Q scores indicated improved outcomes, whereas lower GPSQ scores reflected reduced gender dysphoria. Pre- and post-operative outcomes were compared using paired and unpaired t-tests. Spearman’s correlation coefficients assessed relationships between the parameters assessed.

**Results:**

Significant improvements in post-operative GENDER-Q scores were observed across facial features, with upper face appearance (*r* = 0.54, *p* = 0.001), jaw (*r* = 0.49, *p* = 0.001), and chin (*r* = 0.48, *p* = 0.004), showing greatest gains. GPSQ scores decreased significantly (-7.77 ± 1.61, *p* < 0.001). Pre- to post-operative changes of dysphoria, as measured by the GENDER-Q were associated with overall face (*r* = 0.62, *p* < 0.001), upper face (*r* = 0.64, *p* < 0.001), and chin (*r* = 0.51, *p* = 0.08) score improvement. Furthermore, increased overall facial satisfaction strongly correlated with improvement in satisfaction with the upper face (*r* = 0.54, *p* = 0.001), jaw (*r* = 0.49, *p* = 0.001), and chin (*r* = 0.48, *p* = 0.004).

**Conclusions:**

An individualized approach to gender-affirming facial surgery that prioritizes overall facial harmony, rather than isolated subunit changes, improves gender congruence, facial satisfaction, and alleviates gender dysphoria in transgender patients. In addition, this study advocates for the use of tailored PROMs for the transgender population to more precisely capture and quantify the impact of population-specific interventions and conditions such as gender-affirming surgery and gender dysphoria respectively.

**Supplementary Information:**

The online version contains supplementary material available at 10.1186/s41687-026-01016-1.

## Background

Gender-affirming facial surgery (GAFS) is a collection of procedures that aligns transgender or gender-diverse (TGD) patients’ facial features with their gender identity. The face is a source of significant gender dysphoria for TGD patients, particularly those who were assigned male at birth [1]. GAFS for feminizing procedures includes hairline advancement, frontal sinus setback, brow contouring, brow lifting, rhinoplasty, gonial angle reduction, genioplasty, and chondrolaryngoplasty. Historically, emphasis has been placed on refining the upper facial third to make patients more attractive and feminine [2]. 

While GAFS’s ability to improve gender perception has been demonstrated, a validated patient-reported questionnaire for transgender patients has until recently not been available [3, 4]. Previous patient-reported outcome measures (PROMs) such as PROMIS (Patient-Reported Outcomes Measurement Information System) and FACE-Q PROMs have been used for GAFS patients supporting significant improvement in quality of life and psychosocial wellbeing after surgery [5, 6]. However, the clinical endpoints determined by these PROMs are not specific to this unique patient population [7]. 

The Gender Preoccupation and Stability Questionnaire (GPSQ) is a transgender specific PROM, but does not measure the aesthetic and functional outcomes related to specific gender affirming procedures [8, 9]. Despite its shortcomings, the GPSQ is a population-specific PROM that has been used to measure alleviation of gender dysphoria after gender-affirming care [10]. As such, it is the gold standard PROM for assessment of gender dysphoria.

The GENDER-Q is a novel PROM designed to quantify the effects of the breadth of gender-affirming procedures and treatments specific to the TGD community [11]. The GENDER-Q combines aesthetic, functional, dysphoric, and psychosocial components to create a score that holistically evaluates a patient who has undergone a gender-affirming procedure. In this study as the first of its kind, the GENDER-Q was administered alongside the GPSQ as an assessment of pre- and post-operative outcomes of GAFS to assess the outcomes of gender affirming procedures.

## Methods

### Patient population

Institutional review board approval was obtained prior to study initiation (HIC# 2000031685), and informed consent was secured from all participants.

A total of 100 transfeminine, assigned male at birth (AMAB) patients who were seen in our GAFS clinic between April 2023 and May 2025 received requests to respond to surveys by our group. Eligibility criteria for GAFS at our institution include persistent, well-documented gender dysphoria, capacity for informed consent, and age over 18. Additionally, patients are encouraged to have long-term support from a healthcare professional, such as a therapist or endocrinologist. Although at least one year of GAHT and living in a congruent gender role are strongly advised, neither is mandatory.

GAFS procedures included frontal sinus setback, hairline lowering, brow lift, rhinoplasty, midfacial fat grafting, malar implants, genioplasty, mandibular contouring, and tracheal shave. Although GAFS may be staged, only patients without prior GAFS procedures were included preoperatively; eligible time-points for completing surveys were either prior to initial GAFS or at least 6 months after this surgery for post-operative data collection.

### GENDER-Q

The GENDER-Q overall includes numerous independently functioning scales that cover themes of health-related quality of life, sexual, urination, gender practices, voice, hair, face and neck, body, breasts, genital feminization, chest, genital masculinization, and experience of care [8]. For this study, we included the overall face, upper face, eyebrows, nose, cheeks, lips, chin, jawline, and Adam’s apple scales from the face and neck domain, gender dysphoria and social well-being scales from the health-related quality of life domain and the surgery information scale from the experience of care domain. The overall face section assesses how the individual views their overall facial aesthetics. The facial parts section is comprised of questions evaluating facial subunits, and the facial feature scales asks detailed questions corresponding to specific facial anatomy. The responses for each scale were scored using the Rasch model, which converts raw scores to interval-level data on a scale from 0 to 100. Higher scores indicate better outcomes, such as increased satisfaction with facial appearance or alleviated gender dysphoria. In addition to these scored scales, patients were also asked about time lived in their desired gender and wait time for GAFS.

### Gender preoccupation and stability questionnaire

The Gender Preoccupation and Stability Questionnaire (GPSQ) is a validated tool designed to measure the intensity of gender dysphoria and the degree to which preoccupation with gender identity affects daily life [9]. The GPSQ consists of 14 questions, with a total possible score ranging from 14 to 70. Each question assesses different aspects of gender dysphoria, such as how often it interferes with and disrupts socializing, work, or self-perception. Responses are recorded on a five-point Likert scale, where higher scores indicate worse outcomes.

### Statistical analysis

Paired t-tests compared preoperative and postoperative GENDER-Q and GPSQ scores within the same group. For comparisons between groups with data from only a single time point (either pre-operative or post-operative), unpaired t-tests were applied. Point-biserial correlations were performed between receiving a procedure on a facial part and post-operative satisfaction as well as satisfaction score changes pre- to post-operatively in that facial part. This correlation was conducted for rhinoplasty and nose satisfaction scores, gonial angle reduction and jawline satisfaction, genioplasty and chin satisfaction as well as chondrolaryngoplasty and Adam’s apple satisfaction. Spearman’s rank correlation was used to assess the relationships between GENDER-Q scales, as well as the association between GENDER-Q and GPSQ scores and patient age, follow up time, wait time for surgery and time on HRT. This correlation analysis was performed on both postoperative scores and the changes in scores between pre- and postoperative time points. To control for multiple comparisons, we applied a Bonferroni correction. The significance threshold was adjusted by dividing the standard alpha level (0.05) by the number of comparisons performed (*n* = 13), setting the significance threshold at *p* < 0.00385. Data analysis and visualization were performed using GraphPad Prism 9.4.0 and the Python SciPy library.

## Results

### Participants

In the time course of our study, out of the 100 patients evaluated in clinic who met our criteria, 86 patients (86%) completed the GENDER-Q and 14 refused to initiate or complete the survey (average age 33.2, SD = 8.5). Out of the 86 GENDER-Q survey responders, 34 patients completed the survey pre- and postoperatively (mean follow-up time: 241 days, SD = 101.8), 30 provided only preoperative data due to loss to follow-up, incomplete post-operative survey responses or as they had not yet reached 6 months of follow-up when our study concluded, and 22 provided only postoperative data as they either had incomplete pre-operative survey responses or their pre-operative appointment pre-dated our study (mean follow-up time: 378 days, SD = 252.8). Among these 86 patients, 64 of the patients seen in clinic also completed the GPSQ; 26 of whom did so both prior to and after GAFS, 19 only prior to, and 19 only after surgery.

### Demographics

The surveyed population had an average age of 33.2 years (SD = 8.5), with the majority identifying as Non-Hispanic White (67.4%), followed by Non-White (27.9%) with 4.6% preferring not to answer. A significant proportion (46.5%) had been on gender affirming hormone therapy (GAHT) for 1–3 years, while 4.6% had been on GAHT for less than a year, 16.3% for 3–5 years, 12.8% for 5–10 years, and 12.8% for 10–20 years. Regarding time lived in their desired gender, 2.3% had done so for less than one year, 39.5% for 1 to 3 years, 19.8% for 3 to 5 years, with the rest of our cohort having done so for more than 5 years (38.4%). Approximately half of the patients (51.2%) had not undergone any prior gender-affirming surgeries, with 19.8% reporting previous top surgery, 16.3% bottom surgery and 5.8% top and bottom surgery. Data on the demographic parameters of survey responders is displayed in Table 1.

**Table 1:** Patient demographics (Total of 86 Patients)

Characteristics	Value
**Average age**	33.2 (SD = 8.5)
**Race**	
Non-Hispanic White	58 (67.4%)
Non-White	24 (27.9%)
Preferred Not to Answer	4 (4.6%)
**Years On HRT**	
< 1 year	4 (4.6%)
1–3 years	40 (46.5%)
3–5 years	14 (16.3%)
5–10 years	11 (12.8%)
10–20 years	11 (12.8%)
> 20 years	2 (2.3%)
**Years Living as Desired Gender**	
< 1 year	2 (2.3%)
1–3 years	34 (39.5%)
3–5 years	17 (19.8%)
5–10 years	16 (18.6%)
10–20 years	9 (10.5%)
> 20 years	7 (8.1%)
Prefer not to answer	1 (1.2%)
**Prior gender affirming surgery**	
Top surgery	17 (19.8%)
Bottom surgery	14 (16.3%)
Top and bottom surgery	5 (5.8%)
None	44 (51.2%)
Prefer not to answer	5 (5.8%)

### Procedures received

Regarding the GAFS procedures received, frontal sinus setback was the most performed surgery, completed in 97.1% of the paired cohort and 90.9% of the unpaired postoperative cohort. Other frequently performed procedures included genioplasty (88.2% paired, 77.3% unpaired), and rhinoplasty (79.4% paired, 72.7% unpaired). In both groups, there was an approximate even split between patients who received 4 to 6 of the above procedures and those who received between 7 and 9 procedures. The breakdown of procedures received is shown in Table 2.

**Table 2:** Procedures received among paired cohort (those who completed surveys both pre- and post-operatively) and unpaired (those who completed surveys only post-operatively)

Procedures Received	Paired Cohort	Unpaired Postoperative Cohort
**Frontal sinus setback**	33 (97.1%)	20 (90.9%)
**Hairline lowering**	23 (67.6%)	16 (72.7%)
**Brow lift**	17 (50.0%)	17 (77.3%)
**Supraorbital ridge reduction**	17 (50.0%)	14 (63.6%)
**Midface fat grafting**	27 (79.4%)	15 (68.2%)
**Rhinoplasty**	27 (79.4%)	16 (72.7%)
**Lip lift**	10 (29.4%)	5 (22.7%)
**Gonial angle reduction**	14 (41.2%)	13 (59.1%)
**Genioplasty**	30 (88.2%)	17 (77.3%)
**Chondrolaryngoplasty**	10 (29.4%)	8 (36.4%)
**< 4 Procedures**	0	1 (4.5%)
**4–6 Procedures**	19 (55.8%)	10 (45.4%)
**7–9 Procedures**	15 (44.2%)	11 (50%)
**10 Procedures (All)**	0	0
**Total**	34	22

### T-test comparisons of pre- and post-operative scores

To evaluate the impact of gender-affirming facial surgery (GAFS), paired t-tests were conducted on patients with both preoperative and postoperative GENDER-Q and GPSQ data. Significant improvements were observed across nearly all domains. Postoperative gender dysphoria scores improved significantly (16.45 ± 3.19, *p* < 0.001), along with facial satisfaction, most prominently in the upper face (40.35 ± 5.64, *p* < 0.001), chin (36.76 ± 4.95, *p* < 0.001), nose (31.59 ± 5.55, *p* < 0.001) and jawline. Social well-being scores showed no significant change (0.10 ± 3.02, *p* = 0.976). The GPSQ score decreased significantly (-7.77 ± 1.61, *p* < 0.001), indicating reduced gender dysphoria.

Unpaired t-tests comparing patients with only preoperative and only postoperative data revealed significant postoperative improvements in gender dysphoria (mean difference: 24.52 ± 5.644, *p* < 0.001) and overall facial satisfaction (27.14 ± 4.005, *p* < 0.001). Feature-specific satisfaction was significantly greater in the postoperative group, most saliently for the upper face (47.52 ± 4.826, *p* < 0.001), nose (-45.03 ± 5.271, *p* < 0.001) and jaw (-40.54 ± 5.505, *p* < 0.001). No significant difference was observed in GPSQ scores between unpaired groups (3.632 ± 2.687, *p* = 0.185) or in social well-being (5.530 ± 4.668, *p* = 0.241). Results of both paired and unpaired t-tests are displayed in Table 3.

**Table 3:** Mean of differences and difference of means, from t-tests on GENDER-Q scale scores among paired (those who completed surveys both pre- and post-operatively) and unpaired (those who completed surveys only post-operatively) cohorts

Instrument	Parameter	Paired Cohort	Unpaired Cohort
**GENDER-Q**	**Gender Dysphoria**	16.45 ± 3.19, *p* < 0.001*	24.52 ± 5.644, *p* < 0.001*
	**Social Wellbeing**	0.10 ± 3.02, *p* = 0.976	5.530 ± 4.668, *p* = 0.241
	**Overall Face**	21.62 ± 3.43, *p* < 0.001*	27.14 ± 4.005, *p* < 0.001*
	**Upper Face**	40.35 ± 5.64, *p* < 0.001*	47.52 ± 4.826, *p* < 0.001*
	**Eyebrows**	22.85 ± 3.17, *p* < 0.001*	33.55 ± 5.471, *p* < 0.001*
	**Nose**	31.59 ± 5.55, *p* < 0.001*	45.03 ± 5.271, *p* < 0.001*
	**Cheeks**	16.74 ± 5.63, *p* = 0.005*	35.64 ± 5.754, *p* < 0.001*
	**Lips**	19.09 ± 3.80, *p* < 0.001*	30.29 ± 5.942, *p* < 0.001*
	**Chin**	36.76 ± 4.95, *p* < 0.001*	36.96 ± 5.603, *p* < 0.001*
	**Jaw**	28.15 ± 6.44, *p* < 0.001*	40.54 ± 5.505, *p* < 0.001*
	**Adam’s Apple**	19.65 ± 7.28, *p* = 0.012*	22.21 ± 9.361, *p* < 0.001*
**GPSQ**	**GPSQ Score**	-7.77 ± 1.61, *p* < 0.001*	-3.632 ± 2.687, *p* = 0.185

### Correlation analysis

Point-biserial analysis did not show any significant correlation between receiving a procedure on a facial part and postoperative satisfaction or score change pre- to post-operatively, except for chondrolaryngoplasty, which was associated with improvement in Adam’s apple satisfaction (*r* = 0.59, *p* < 0.001) **(**Table 4**)**.

**Table 4:** Point-biserial correlations between receipt of procedure on facial part, postoperative satisfaction and score changes in the corresponding GENDER-Q scale

	Postoperative Score		Score Change
**Rhinoplasty and Nose Satisfaction**	*r*=-0.08, *p* = 0.511	*r = 0.17, p = 0.332*	
**Genioplasty and Chin Satisfaction**	*r*=-0.08, *p* = 574	*r*=-0.09, *p* = 0.626	
**Gonial Angle Reduction and Jaw Satisfaction**	*r*=-0.20, *p* = 0.132	*r*=-0.28, *p* = 0.102	
**Chondrolaryngoplasty and Adam’s Apple Satisfaction**	*r* = 0.12, *p* = 0.385	*r* = 0.59, *p* < 0.001*	

The results of the Spearman correlation analysis revealed significant associations between postoperative GENDER-Q scale scores. The Spearman analysis of postoperative scores also detected statistically significant correlations across GENDER-Q scales. Dysphoria significantly correlated with social wellbeing (*r* = 0.77, *p* < 0.001) and overall face (*r* = 0.55, *p* < 0.001), jaw (*r* = 0.47, *p* < 0.001) and upper face (*r* = 0.45, *p* < 0.001). Social well-being and overall face (*r* = 0.57, *p* < 0.001), jaw (*r* = 0.55, *p* < 0.001) and upper face (*r* = 0.50, *p* < 0.001) scores were also significantly associated. Overall facial satisfaction was positively correlated with all facial feature scores, most significantly so with upper face (*r* = 0.79, *p* < 0.001), nose (*r* = 0.76, *p* < 0.001) and jaws (*r* = 0.74, *p* < 0.001).

**Fig. 1:**
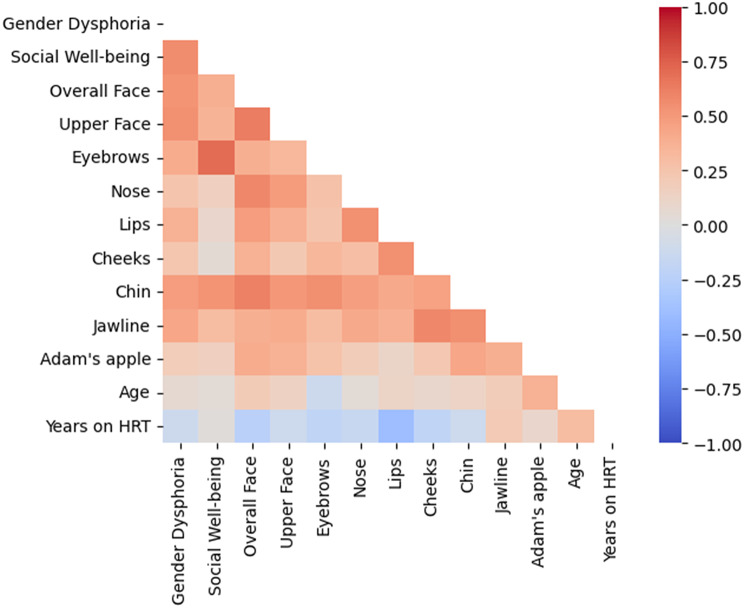
Spearman correlation heatmap between pre- to post-operative changes in GENDER-Q scale scores and patient-related parameters (Age and Time on HRT)

Postoperative facial feature satisfaction scores also significantly correlated with each other. Other parameters such as age, years on HRT and GPSQ scores, did not correlate with GENDER-Q post-operative scores. The heatmap corresponding to this Spearman analysis is Fig. 1.

**Fig. 2:**
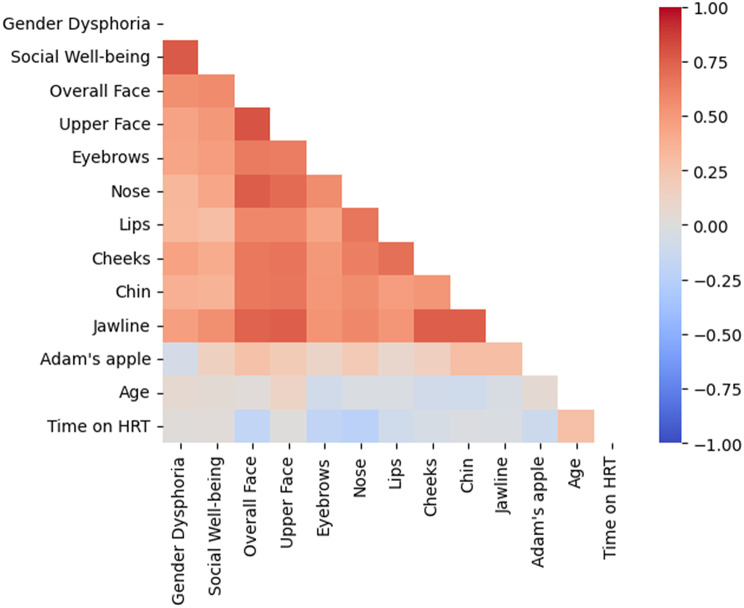
Spearman correlation heatmap between postoperative GENDER-Q scale scores and patient-related parameters (Age and Time on HRT)

As for pre- to post-operative score changes correlation; change of dysphoria score at this time-point showed strong positive correlation with social well-being improvement (*r* = 0.56, *p* < 0.001), overall face satisfaction (*r* = 0.53, *p* < 0.001), upper face appearance (*r* = 0.54, *p* = 0.001), and chin (*r* = 0.48, *p* = 0.0037). Correlations between social well-being and various facial features were also substantial. The eyebrows (*r* = 0.697, *p* < 0.001) and chin (*r* = 0.525, *p* < 0.001) satisfaction scores were most significantly associated with social satisfaction. Changes in overall face satisfaction was positively associated with higher satisfaction with every facial feature, and significantly associated with all facial parts, most significantly with upper face (*r* = 0.63, *p* < 0.001), chin (*r* = 0.61, *p* < 0.001) and nose (*r* = 0.58, *p* < 0.001). GPSQ scores, time on HRT and age did not correlate significantly with each other or other variables. Results of these Spearman correlations are shown in the Fig. 2 heatmap, both correlations are further detailed in Tables 1 and 2, Supplementary Digital Content 1.

## Discussion

We present the first study to evaluate GAFS using the GENDER-Q, a novel population-specific PROM for evaluation of gender surgery outcomes, and the GPSQ, the present gold standard for gender dysphoria. Ours is the first study to implement any validated measure to evaluate improvement in gender dysphoria after GAFS. Our study demonstrates that GAFS positively impacts multiple facets of TGD patients’ lives, contributing to overall improvements in gender dysphoria, patients’ perceived gender congruence, and overall facial aesthetics among the TGD population. These findings align with prior research suggesting that GAFS plays a critical role in alleviating gender dysphoria in the TGD population [5, 6]. 

The GENDER-Q and GPSQ allowed our team to capture nuances not typically addressed by PROMs presently used in the gender-affirming surgery literature [7]. Prior studies have used proxy measures for patients’ experience of gender dysphoria, including depression scoring and online third-party surveys [12, 13]. By capturing metrics relevant to social well-being, body-gender congruence, and dysphoria, our study offers a more comprehensive assessment of GAFS outcomes. These findings underscore the importance of including GAFS in gender-affirming care, as it remains under-covered by insurance in the United States [14, 15]. Our data showing GAFS’s role in alleviating gender dysphoria supports insurance coverage for these procedures, and greater accessibility of GAFS for TGD patients.

In reviewing the Spearman analysis, our findings validate prior research with the identification of a positive correlation in both upper and lower face aesthetics and gender dysphoria alleviation. The upper face, particularly the brow, has long been considered the most impactful area of facial feminization surgery. However, our study shows that improvements in lower facial aesthetics such as chin and jawline correlated strongly with alleviation of TGD patients’ gender dysphoria and improvement of social satisfaction [2]. Moreover, there was a positive correlation in overall facial aesthetics for every gender affirming facial procedure performed, as shown in Fig. 1. Our data show that dysphoria and overall facial aesthetic outcomes are not specifically attributed to individual facial subunits. Rather, we have shown the importance of developing facial harmony using an individualized patient-specific approach.

Alper et al. utilized facial recognition software to show how GAFS leads to improved gender congruence. However, the study did not identify whether improved gender congruence led to improved patient outcomes [3]. The GENDER-Q’s facial aesthetic subsection includes this nuance in its calculated scores, and through our Spearman correlation, our team was able to demonstrate that improvements in overall facial aesthetics correlated positively with improvements in social wellbeing and alleviation of gender dysphoria – further highlighting the importance of achieving facial harmony. Of note, though the GENDER-Q and GPSQ are validated tools to evaluate gender dysphoria in the TGD community, their respective gender dysphoria scores did not correlate in our study. However, this could be due to the scaling of each of the instruments. Further, the smaller scale and lower power in our unpaired group likely contributed to the lack of significance in pre- and post-operative GPSQ scores.

Pre- and post-surgical comparisons indicate that despite a positive correlation between social well-being and higher facial aesthetic scores, social well-being subunit scores did not significantly improve postoperatively in either cohort. This could be due to the character of the questions in the GENDER-Q social wellbeing subsection. They focus strongly on how the patient perceives the way that they are treated by others. Our findings suggest that though GAFS provides our TGD patients with personal improvement, no surgical intervention can make up for experienced discrimination or paucity of social support. For additional context and comparison, studies using the FACE-Q in facial aesthetic populations have demonstrated significant improvements in facial satisfaction, as well as psychological and social well-being, following both surgical and minimally invasive treatments. Notably, the HARMONY study showed that a multimodal approach to facial rejuvenation was associated with substantial gains in psychological well-being and social confidence, with effect sizes comparable to those reported after surgical procedures. These findings highlight the limitations of clinician-rated aesthetic measures alone and underscore the value of validated PRO instruments in capturing psychosocial outcomes that are meaningful to patients. Collectively, evidence supports the conceptualization of facial interventions as having multidimensional impacts that extend beyond physical appearance, reinforcing the importance of patient-centered outcome assessment [16, 17]. 

With respect to our cohort, we found that around 5% of patients were either on GAHT for less than 1 year or chose not to undergo GAHT in congruence with their gender expression. These patients all met criteria for proceeding with gender affirming facial surgery including well documented gender dysphoria and capacity to provide informed consent.

Our findings are limited by sample size and the single-center nature of the research. Future studies could benefit from a multicenter approach with larger populations to confirm these findings across various demographics and surgical techniques. Additionally, longer-term longitudinal studies examining the impact of GAFS on mental health, social integration, and overall quality of life would provide valuable information on the sustained benefits and potential challenges associated with GAFS.

## Conclusions

This study highlights the significant, positive impact of GAFS on gender dysphoria, gender congruence of our patients’ facial features, and overall facial harmony among the TGD population. The use of the GENDER-Q and GPSQ allowed for a nuanced understanding of how GAFS meets unique patient needs, contributing to a holistic approach in evaluating outcomes of gender-affirming surgery. The questionnaires also show that patient outcomes are not beholden to individual facial subunits, and rather are the result of improved facial harmony based on patient-specific needs. These findings add to the growing body of literature supporting GAFS as an essential component of transgender health care, emphasizing its role in alleviating gender dysphoria and improving gender-congruent aesthetics. Future studies continuing population specific PROMs with broader populations and longer follow-up periods are recommended to further validate these findings and ensure that all TGD individuals have access to comprehensive, affirming, and effective healthcare options.

## Supplementary Information

Below is the link to the electronic supplementary material.

Supplementary Material 1

## Data Availability

The datasets used and analyzed during the current study are available from the corresponding author on reasonable request.

## Abbreviations

GAFS: Gender-Affirming Facial Surgery
GENDER-Q: Gender-Q Questionnaire
PROM: Patient-Reported Outcome Measure
GPSQ: Gender Preoccupation and Stability Questionnaire
TGD: Transgender and Gender-Diverse
PROMIS: Patient-Reported Outcomes Measurement Information System
AMAB: Assigned Male At Birth
GAHT: Gender-Affirming Hormone Therapy
HRT: Hormone Replacement Therapy
SD: Standard Deviation
