# Supplementary material for: Patient satisfaction following gender affirming facial surgery: a GENDER-Q and gender preoccupation and stability questionnaire study (GPSQ)
Source: J Patient Rep Outcomes. 2026 May 4;10:106. doi: 10.1186/s41687-026-01016-1 (PMC13315363; doi:10.1186/s41687-026-01016-1)
Supplement: Supplementary file 1 — Supplementary Material 1 [file 41687_2026_1016_MOESM1_ESM.docx]

|  | Gender Dysphoria | | | Social Well-being | | Overall Face | | Upper Face | | Eyebrows | | Nose | | Lips | | Cheeks | | Chin | | Jawline | | Adam's apple | | Age |
| --- | --- | --- | --- | --- | --- | --- | --- | --- | --- | --- | --- | --- | --- | --- | --- | --- | --- | --- | --- | --- | --- | --- | --- | --- |
| Social Well-being | | **0.77** |  | |  | |  | |  | |  | |  | |  | |  | |  | |  | |  | |
| Overall Face | | **0.55** | **0.57** | |  | |  | |  | |  | |  | |  | |  | |  | |  | |  | |
| Upper Face | | **0.45** | **0.50** | | **0.79** | |  | |  | |  | |  | |  | |  | |  | |  | |  | |
| Eyebrows | | **0.44** | **0.48** | | **0.64** | | **0.63** | |  | |  | |  | |  | |  | |  | |  | |  | |
| Nose | | 0.34 | **0.43** | | **0.76** | | **0.71** | | **0.56** | |  | |  | |  | |  | |  | |  | |  | |
| Lips | | 0.33 | 0.29 | | **0.58** | | **0.58** | | **0.44** | | **0.66** | |  | |  | |  | |  | |  | |  | |
| Cheeks | | **0.46** | 0.40 | | **0.65** | | **0.67** | | **0.50** | | **0.62** | | **0.68** | |  | |  | |  | |  | |  | |
| Chin | | **0.38** | 0.36 | | **0.65** | | **0.66** | | **0.51** | | **0.56** | | **0.48** | | **0.52** | |  | |  | |  | |  | |
| Jawline | | **0.47** | **0.55** | | **0.74** | | **0.75** | | **0.53** | | **0.58** | | **0.52** | | **0.75** | | **0.75** | |  | |  | |  | |
| Adam's apple | | -0.07 | 0.15 | | 0.27 | | 0.20 | | 0.11 | | 0.21 | | 0.09 | | 0.16 | | 0.29 | | 0.29 | |  | |  | |
| Age | | 0.07 | 0.05 | | 0.03 | | 0.13 | | -0.09 | | -0.03 | | -0.03 | | -0.09 | | -0.10 | | -0.04 | | 0.06 | |  | |
| Time on HRT | | 0.02 | 0.03 | | -0.18 | | 0.01 | | -0.19 | | -0.23 | | -0.10 | | -0.05 | | -0.02 | | -0.03 | | -0.12 | | 0.28 | |

Table 1. Spearman Correlation of Post-Operative GENDER-Q Scale Scores and Patient-Related Parameters: R values.

Bold numbers represent statistically significant values.

|  | Gender Dysphoria | | | Social Well-being | | Overall Face | | Upper Face | | Eyebrows | | Nose | | Lips | | Cheeks | | Chin | | Jawline | | Adam's apple | | Age |
| --- | --- | --- | --- | --- | --- | --- | --- | --- | --- | --- | --- | --- | --- | --- | --- | --- | --- | --- | --- | --- | --- | --- | --- | --- |
| Social Well-being | | **0.56** |  | |  | |  | |  | |  | |  | |  | |  | |  | |  | |  | |
| Overall Face | | **0.53** | 0.39 | |  | |  | |  | |  | |  | |  | |  | |  | |  | |  | |
| Upper Face | | **0.54** | 0.36 | | **0.63** | |  | |  | |  | |  | |  | |  | |  | |  | |  | |
| Eyebrows | | 0.40 | **0.70** | | 0.39 | | 0.33 | |  | |  | |  | |  | |  | |  | |  | |  | |
| Nose | | 0.25 | 0.16 | | **0.58** | | **0.49** | | 0.27 | |  | |  | |  | |  | |  | |  | |  | |
| Lips | | 0.37 | 0.10 | | 0.48 | | 0.38 | | 0.25 | | **0.54** | |  | |  | |  | |  | |  | |  | |
| Cheeks | | 0.24 | 0.05 | | 0.37 | | 0.22 | | 0.34 | | 0.29 | | **0.54** | |  | |  | |  | |  | |  | |
| Chin | | 0.48 | **0.53** | | **0.61** | | **0.51** | | **0.55** | | 0.47 | | 0.42 | | 0.46 | |  | |  | |  | |  | |
| Jawline | | 0.43 | 0.30 | | 0.39 | | 0.40 | | 0.30 | | 0.42 | | 0.38 | | **0.58** | | **0.55** | |  | |  | |  | |
| Adam's apple | | 0.18 | 0.16 | | 0.40 | | 0.37 | | 0.26 | | 0.19 | | 0.11 | | 0.23 | | 0.43 | | 0.39 | |  | |  | |
| Age | | 0.07 | 0.04 | | 0.20 | | 0.14 | | -0.12 | | 0.04 | | 0.12 | | 0.09 | | 0.13 | | 0.19 | | 0.37 | |  | |
| Time on HRT | | -0.12 | 0.02 | | -0.24 | | -0.11 | | -0.20 | | -0.15 | | -0.40 | | -0.20 | | -0.11 | | 0.21 | | 0.10 | | 0.30 | |

Table 2. Spearman Correlation of Changes in GENDER-Q Scale Scores and Patient-Related Parameters: R values.

*Asterisks represent statistically significant values.
